# Characterization of the CD14^++^CD16^+^ Monocyte Population in Human Bone Marrow

**DOI:** 10.1371/journal.pone.0112140

**Published:** 2014-11-04

**Authors:** Manuela Mandl, Susanne Schmitz, Christian Weber, Michael Hristov

**Affiliations:** 1 Institute for Cardiovascular Prevention, Ludwig-Maximilians-University (LMU), Munich, Germany; 2 Munich Heart Alliance, Munich, Germany; University of York, United Kingdom

## Abstract

Numerous studies have divided blood monocytes according to their expression of the surface markers CD14 and CD16 into following subsets: classical CD14^++^CD16^−^, intermediate CD14^++^CD16^+^ and nonclassical CD14^+^CD16^++^ monocytes. These subsets differ in phenotype and function and are further correlated to cardiovascular disease, inflammation and cancer. However, the CD14/CD16 nature of resident monocytes in human bone marrow remains largely unknown. In the present study, we identified a major population of CD14^++^CD16^+^ monocytes by using cryopreserved bone marrow mononuclear cells from healthy donors. These cells express essential monocyte-related antigens and chemokine receptors such as CD11a, CD18, CD44, HLA-DR, Ccr2, Ccr5, Cx3cr1, Cxcr2 and Cxcr4. Notably, the expression of Ccr2 was inducible during culture. Furthermore, sorted CD14^++^CD16^+^ bone marrow cells show typical macrophage morphology, phagocytic activity, angiogenic features and generation of intracellular oxygen species. Side-by-side comparison of the chemokine receptor profile with unpaired blood samples also demonstrated that these rather premature medullar monocytes mainly match the phenotype of intermediate and partially of (non)classical monocytes. Together, human monocytes obviously acquire their definitive CD14/CD16 signature in the bloodstream and the medullar monocytes probably transform into CD14^++^CD16^−^ and CD14^+^CD16^++^ subsets which appear enriched in the periphery.

## Introduction

Current knowledge defines three major monocyte subsets in human peripheral blood based on the expression patterns of CD16 (FcγRIII) and the LPS-receptor CD14: classical CD14^++^CD16^−^, intermediate CD14^++^CD16^+^ and nonclassical CD14^+^CD16^++^ monocytes [1]. These subsets differ essentially in their chemokine receptor expression, phagocytic activity and tissue distribution during inflammation or steady-state conditions [2]. Hence, the monocyte subsets are differentially involved in the pathophysiology of inflammation, atherosclerosis and regeneration after injury [3]–[5]. In particular, the unique features of the intermediate in contrast to the nonclassical subset (both previously referred as CD16^+^ monocytes) has become recently more evident and especially in cardiovascular disease this subset was shown to predict independently cardiovascular events at follow-up [6]–[8]. Thus, human monocyte subsets may represent a novel prognostic marker or therapeutic targets in clinical medicine and detailed investigation of their characteristics in bone marrow (BM) as compared to peripheral blood appears important for better understanding of the subset-specific development, maturation and functional specialization. However, the experimental research on resident human monocytes in BM together with the plasticity of subset development and trafficking to the periphery is highly restricted and only few published data exist. Therefore the aim of our study is to provide novel evidence on medullar CD14/CD16 monocytes by using commercially available cryopreserved BM samples from healthy donors as an alternative to fresh BM and to reveal further the effects of freezing/thawing on monocyte number, function and chemokine receptor expression.

## Materials and Methods

### Cells

Cryopreserved bone marrow mononuclear cells (BMCs; 25×10^6^ or 100×10^6^) from ten healthy single donors were obtained by Lonza (Cologne, Germany). The BMCs were thawed and maintained overnight prior all further experiments in *Iscove's Modified Dulbecco's Medium* (IMDM; PAA, Pasching, Austria) supplemented with 10% FCS and antibiotics in 24-well plates (1×10^6^ cells per well). After written informed consent was obtained, 20 ml of citrate anticoagulated peripheral blood were collected by aseptic venipuncture from ten healthy volunteers. The procedure was approved by the Ethics Committee of the Medical Faculty of the LMU Munich. PBMCs were separated by *Biocoll* (Biochrom AG, Berlin, Germany) density gradient centrifugation. Some fresh PBMCs were immediately analyzed by flow cytometry as described below while the remaining cells were frozen in RPMI 1640 medium (PAA) with 10% FCS and 5% dimethyl sulfoxide (DMSO; Sigma-Aldrich, Taufkirchen, Germany). The cells were cooled to −80°C in a *CoolCell Freezing Container* (BioCision, Mill Valley, CA) before being transferred to liquid nitrogen for storage. The cryopreserved PBMCs were thawed/cultured after one week of storage in the same fashion as the BMCs.

### Flow cytometry analysis of surface antigens

Cells were collected by thorough resuspension and the flow cytometry analysis was performed as 4-color experiment by using the *Lyoplate Screening Panel* (BD, Heidelberg, Germany) following staining with anti-human CD45-PerCP (clone 2D1; BD), CD14-FITC (clone HCD14) and CD16-PE (clone 3G8) mAbs (both Biolegend, Fell, Germany). The control tube included anti-human CD45/CD14/CD16 mAbs and the AlexaFluor 647-conjugated secondary Ab of the screening panel. Further anti-human mAbs used for multicolor flow cytometry were as follows: CD117-APC (clone 104D2), CD16-Pacific Blue (clone 3G8), Ccr2-APC mouse IgG2a (clone K036C2, 100 µg/ml), Ccr5-AlexaFluor 647 rat IgG2a (clone HEK/1/85a, 100 µg/ml), Cx3cr1-AlexaFluor 647 rat IgG2b (clone 2A9-1, 100 µg/ml), Cxcr2-PE mouse IgG1 (clone 5E8, 50 µg/ml), Cxcr4-PE mouse IgG2a (clone 12G5, 50 µg/ml; all Biolegend); CD15-eFluor450 (clone HI98) and CD56-APC (clone MEM188; both eBioscience, Vienna, Austria); Tie2-APC (clone 83715; R&D Systems, Wiesbaden, Germany). Matching fluorescent isotype control Abs (clones MOPC-21/173 and RTK2758/4530; Biolegend) were used at same concentration as the respective mAb. Sample tubes were acquired on a FACSCanto II with Diva v8.0 software (BD) and appropriate CS&T bead calibration setting. The fluorescence compensation was automatically calculated using *OneComp eBeads* (eBioscience). At least 10.000 events were recorded within the target population. The specific mean fluorescence intensity (MFI) was assessed by subtracting the background of the respective isotype control. To determine chemokine receptor expression over time some BMCs were cultured in RPMI 1640 medium with 10% FCS and antibiotics on 6-well plates (4×10^6^ cells per well). The flow cytometry analysis for expression of CD45, CD14, CD16, Ccr2, Ccr5 and Cx3cr1 was performed after one and seven days of culture as described above.

### Functional assays

Single-donor BMCs (10^7^) or pooled BMCs (75×10^6^) of three individual donors were sorted (FACSAria III; BD) after staining for CD14/CD16/CD45 and plated on 24-well plates or 4-well chamber slides in RPMI 1640 medium with 10% FCS and antibiotics. Phagocytosis of FITC-conjugated latex beads (1∶1000; Sigma-Aldrich) and hydroethidine (10 µg/ml; Invitrogen, Darmstadt, Germany) staining for spontaneous intracellular superoxide production in PBMC- or sorted BMC-derived macrophages were determined by flow cytometry after 24 hours in culture. Cytoskeletal filaments in cultured BMCs were visualized by fluorescence microscopy (Leica DM6000B, Wetzlar, Germany) after cell fixation/permeabilization (*Cytofix/Cytoperm*; BD) following incubation with anti-α-tubulin-FITC (Sigma-Aldrich) mAb and rhodamine-phalloidin (Biotium, Hayward, CA). *Vectashield* with DAPI (Vector Laboratories, Peterborough, UK) was used as mounting medium and to stain cell nuclei. In another experimental setup the sorted BMCs were cultured in angiogenesis μ-slide (IBIDI, Martinsried, Germany) on *Matrigel* (BD) supplemented with 50 ng/ml VEGF and 100 ng/ml Cxcl12 (both from Peprotech, Hamburg, Germany).

### Statistics

Data were analyzed using *Prism 5* software (GraphPad Inc., La Jolla, CA) and presented as mean±SD. Data distribution was assessed by the *Shapiro-Wilk* normality test. Accordingly, we have used for comparison between two groups the unpaired *t test* or nonparametric *Mann-Whitney* test and for comparison between three groups one-way ANOVA with *Newman-Keuls* post-test. Differences with *p*<0.05 were considered statistically significant.

## Results

**Figure 1:**
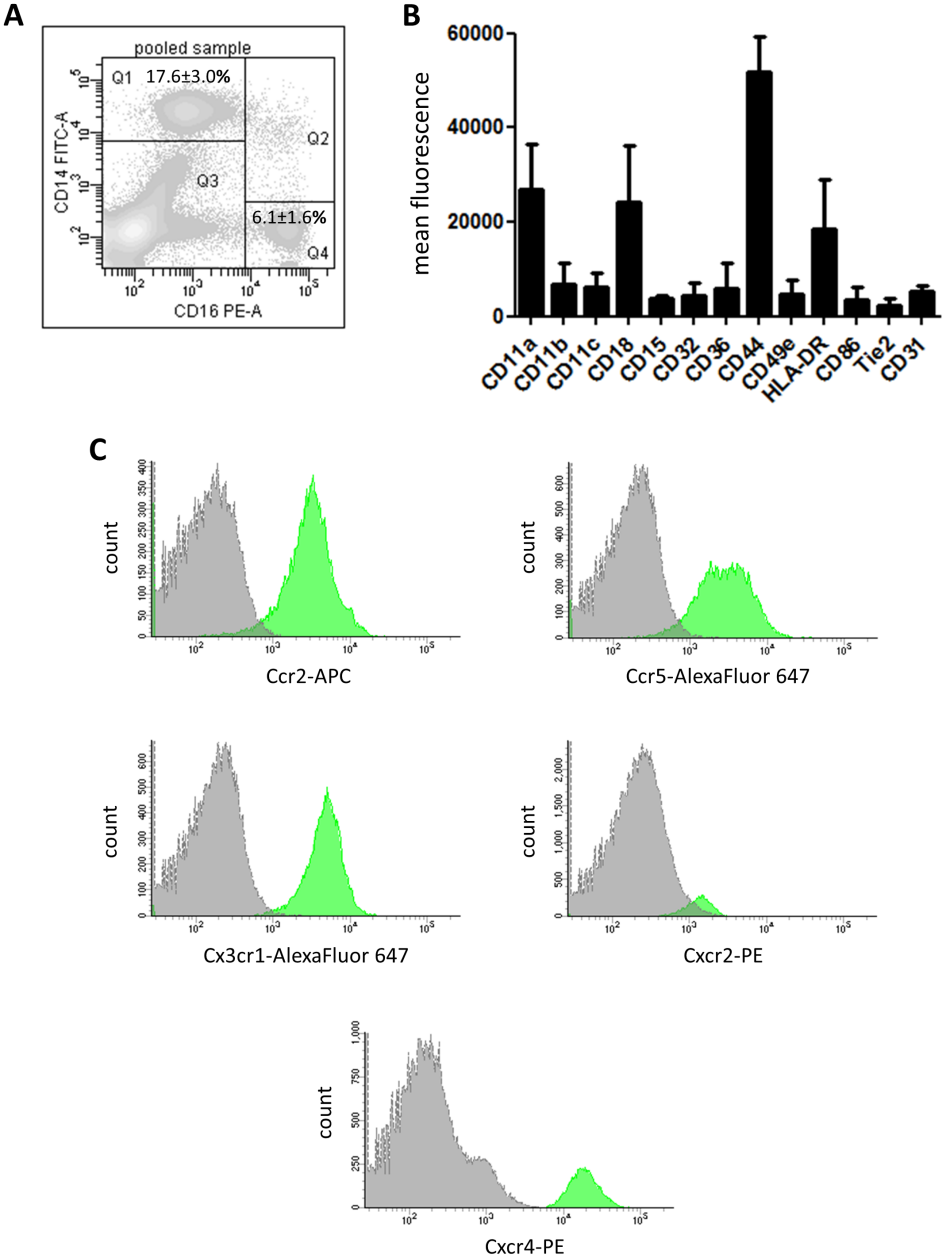
Characterization of CD14^++^CD16^+^ bone marrow monocytes by flow cytometry. (A) Representative contour plot of pooled BMC sample from three single donors showing a clear separation of two populations: CD14^++^CD16^+^ (Q1) and CD14^−^CD16^++^ (Q4). Gating was performed first in FSC/SSC dot plot following gating on CD45^+^ events in a SSC/CD45 dot plot. Finally, the expression of CD14 and CD16 was evaluated inside the CD45^+^ population. (B,C) Expression of monocyte-related antigens and representative histogram overlays with isotype control (gray filled) of crucial chemokine receptors on CD14^++^CD16^+^ BMCs as analyzed by flow cytometry. Data are from 3 to 6 individual donors.

Flow cytometry analysis of thawed human BMCs after staining for CD14, CD16 and CD45 showed clear separation of two scatter populations (Fig. 1A): a larger CD14^++^ population (Q1: 17.6±3.0% of CD45^+^ cells) with low-to-intermediate expression of CD16 (CD16^+^) and a smaller CD14^−^CD16^++^ population (Q4: 6.1±1.6% of CD45^+^ cells). The CD14^−^CD16^++^ cells in Q4 were also CD15^−^CD56^++^ thus probably referring to NK and NK-T cells (data not shown).

Further analysis of the CD14^++^CD16^+^ population revealed expression of CD11a, CD18, CD44 and HLA-DR together with other essential monocyte-related antigens (Fig. 1B;S1). There was also expression of the chemokine receptors Ccr2, Ccr5, Cx3cr1, Cxcr2 and Cxcr4 as shown in Fig. 1C. Moreover, the expression of Ccr2 on medullar macrophages was significantly up-regulated after 7 days in culture (Table 1).

**Table 1:** Chemokine receptor expression on cultured medullar macrophages from six single donors as analyzed by flow cytometry.

Receptor	1 day	7 days	*p-value*
**Ccr2**	97±29%	351±192%	*0.04*
**Ccr5**	152±31%	340±136%	*0.06*
**Cx3cr1**	113±41%	328±159%	*0.06*

The MFI values of CD14^++^CD16^+^ monocytes before plating served as control ( = 100%).

**Figure 2:**
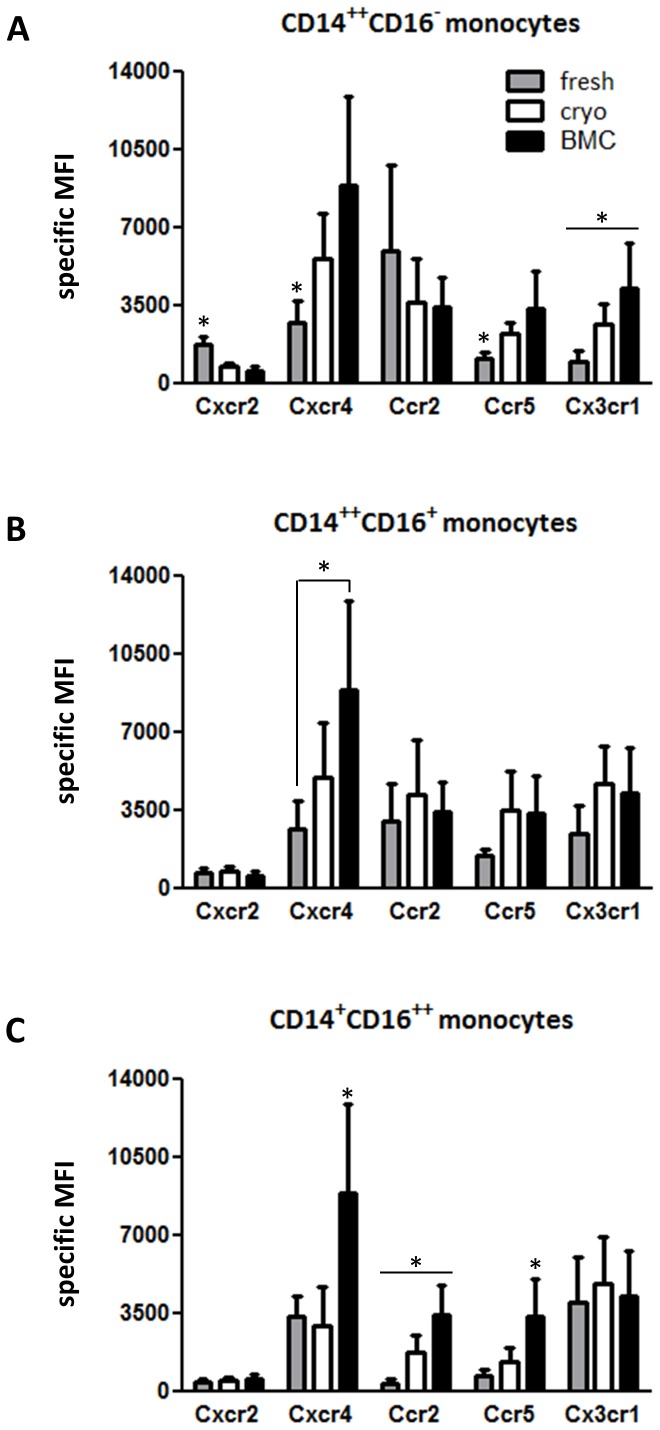
Side-by-side comparison of chemokine receptor expression on medullar with frozen or fresh blood monocytes. PBMCs from different donors were separated by density gradient centrifugation. Chemokine receptor expression was analyzed and compared on fresh monocyte subsets (gray bars) versus cryopreserved cells of the same donor (open bars) or unpaired medullar monocytes (black bars). *p<0.05, n = 6–10.

Side-by-side examination of chemokine receptor expression on CD14^++^CD16^+^ BM monocytes versus unpaired cryopreserved blood samples showed very similar levels for Ccr2, Ccr5 and Cx3cr1 as compared to intermediate monocytes together with almost identical levels of Ccr2 on classical and Cx3cr1 on nonclassical monocytes (Fig. 2A–C; Table S1). Further comparison with unpaired fresh blood monocytes revealed identical expression of Cx3cr1 as to the nonclassical subset (Fig. 2C). Overall, the BM monocytes showed highest levels for Cxcr4 (Fig. 2A–C) and more than 20× higher expression of the hematopoietic precursor marker CD117 (data not shown) whereas fresh classical monocytes had highest levels of Cxcr2 and Ccr2 (Fig. 2A–C). Another analysis of paired fresh versus cryopreserved blood monocytes demonstrated significant decrease of Cxcr2 next to increased expression of Cxcr4, Ccr5 and Cx3cr1 on classical monocytes while thawed nonclassical monocytes had higher expression only of Ccr2 as compared to freshly processed cells (Fig. 2A,C). Finally, cryopreservation failed to affect the percentages of all blood monocyte subsets (Table 2).

**Table 2:** Percentages of blood monocyte subsets before and after freezing/thawing (n = 7–10).

Subset	fresh	thawed	*p-value*
**CD14^++^CD16^−^**	80.1±7.0%	82.8±3.0%	*0.54*
**CD14^++^CD16^+^**	3.7±2.0%	3.8±1.5%	*0.96*
**CD14^+^CD16^++^**	6.2±2.8%	6.3±1.7%	*0.93*

**Figure 3:**
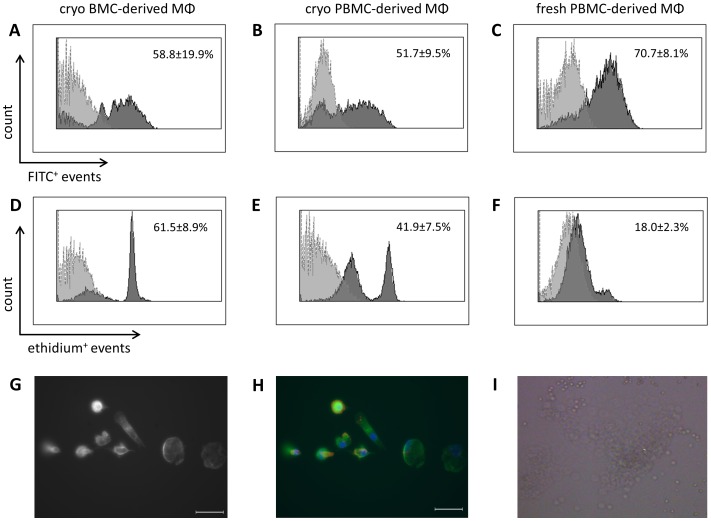
Functional analyses of CD14^++^CD16^+^ BMC- and PBMC-derived macrophages. Representative flow cytometry histogram overlays for uptake of FITC-conjugated beads (A–C) and hydroethidine staining (D–F) in macrophages cultured from CD14^++^CD16^+^ BMCs, fresh or cryopreserved PBMCs of three single donors. The gray filled histogram shows cells without beads or dye, respectively. (G,H) Phase contrast and immunofluorescent overlay of CD14^++^CD16^+^ BMC-derived macrophages which were cultured in chamber slides and stained for α-tubulin-FITC/rhodamine-phalloidin after 7 days as described in methods. Cell nuclei were counterstained with DAPI. The scale bar indicates 40 µm. (I) Cluster formation and shape change of CD14^++^CD16^+^ BM cells on Matrigel after 72 hours. Data in (G–I) are representative images of triplicates from pooled sample of three independent donors.

Functional analysis demonstrated phagocytic activity by 58.8±19.9% of sorted CD14^++^CD16^+^ BMCs as well as spontaneous generation of reactive oxygen species (ROS) at 61.5±8.9% after 24 hours in culture (Fig. 3A,D). By comparison, macrophages originating from frozen PBMCs showed phagocytic capacity of 51.7±9.5% and spontaneous superoxide production at 41.9±7.5% (Fig. 3B,E) while freshly isolated macrophages revealed 70.7±8.1% of bead uptake and 18.0±2.3% of ROS production after 24 hours of culture (Fig. 3C,F). Microscopic immunofluorescence analysis of cultured CD14^++^CD16^+^ BMCs further confirmed typical *fried egg-like* macrophage morphology with α-tubulin/F-actin-rich protrusions (Fig. 3G,H). The CD14^++^CD16^+^ BMCs displayed also angiogenic properties as they express CD31, Tie2 and Cxcr4, and fuse to clusters with shape change in Matrigel (Fig. 1B,C;3I).

## Discussion

To our best knowledge, this is the first study characterizing CD14/CD16 monocyte subsets in cryopreserved human BM of healthy donors as a possible alternative to freshly prepared cells. We found a major pool of CD14^++^CD16^+^ BMCs that seems to correspond to intermediate blood monocytes. These cells clearly feature monocytes/macrophages as illustrated by their phenotype and function. Another important analysis mapped the effect of freezing/thawing on chemokine receptor expression, subset proportion and function of bloodstream monocytes. A limitation of our study is the lack of direct validation and comparison with fresh marrow samples, ideally obtained from the same donor. However, in contrast to venous blood it is not conceivable to collect routinely fresh BM of healthy volunteers for experimental research. Consequently, only one prior work has evaluated the immunophenotype of monocyte subsets in BM aspirates originating from healthy individuals with suspected peripheral lymphomas up to now [9].

Compared to humans, the recruitment of mouse monocyte subsets *in vivo* was investigated in detail by using several models of receptor knock-out, BM transplantation, cell depletion or adoptive transfer [10]–[12]. Up to now, there are two major murine monocyte subsets: the Gr1^+^/Ly6C^hi^ cells are similar to human CD14^++^CD16^−^ monocytes and Gr1^−^/Ly6C^low^ cells are considered as counterparts of the CD14^+^CD16^++^ monocytes [3], [10], [11]. During egress from BM, the CC-chemokine Ccl2 (MCP-1) plays a central role by either inducing Ccr2-dependent chemokinesis or chemotaxis of Ccr2^+^Ly6C^hi^ mouse monocytes [11]. Our data demonstrated comparable expression levels of Ccr2 between medullar and thawed CD14^++^ blood monocytes. Remarkably, we found higher amount of Cxcr4 on BM monocytes in comparison with frozen or fresh bloodstream monocytes. As human CD14^++^CD16^+^ BMCs express also the adhesion molecule CD44 they may be accessorily attracted by ligands for CD44 and Cxcr4 such as glycosaminoglycans and Cxcl12 to move towards the vascular niche of the BM and to enter the bloodstream. According to their origin BM monocytes still express the precursor marker CD117 which was absent on their terminally differentiated counterparts in the bloodstream. Further analysis of chemokine receptors revealed very similar expression profile between medullar and frozen intermediate monocytes. Fresh classical monocytes showed highest levels of Ccr2 while the expression of Cx3cr1 was similar between medullar and CD16^++^ blood subsets (fresh or frozen). Of note, the expression of Ccr2 on BMC-derived macrophages was inducible in culture. Thus, human monocytes probably acquire their differential chemokine receptor signature (e.g. high levels of Ccr2 on the classical subset) during or after recruitment to the periphery with simultaneous decline of Cxcr4 and CD117 in terms of differentiation. It is well conceivable that such maturation may occur more rapid *in vivo* than *in vitro*, especially during inflammatory conditions or infection with accompanying monocytosis and preferential shift of classical monocytes.

Our results next reveal higher expression of some chemokine receptors on blood monocytes shortly after thawing while the ratio of receptor expression together with subset frequencies remains unaffected as compared to fresh monocytes. Accordingly, cryopreservation may affect at least transiently the corresponding chemokine receptors on BMCs as well. Furthermore, macrophages that originated from frozen blood or BM monocytes showed similar phagocytic capacity but had significantly increased ROS production as compared to freshly obtained blood macrophages. One possible explanation for this difference could be transient cell activation following cryopreservation. In line with our findings previous studies have already reported that cryopreservation can rather influence activation status than frequency or function of mononuclear cells. In particular, human T-cells, monocytes or circulating angiogenic cells can be thawed without considerable alteration of their phenotype and function [13]–[15]. Cryopreservation is also routinely used for storage of autologous CD34^+^ cells suggesting that cryopreserved blood cells usually match fine fresh cells [16]. Hereby, even if the cryopreserved cells in our study were not fully equal in phenotype and ROS generation to fresh cells (at least shortly after thawing) they may represent another alternative in terms of availability, storage, transportation and functionality.

Surprisingly, we also found that nonclassical monocytes do not certainly persist as population with clear CD14/CD16 boundaries among thawed BMCs. As mentioned above it is rather unlike that cryopreservation *per se* could impact the abundance of nonclassical monocytes since their proportion remains well-preserved after freezing/thawing as confirmed also by others [17]. As further shown in a previous study, the nonclassical monocytes in fresh marrow samples were not primary identified as separate CD14^+^CD16^++^ scatter population but first on the basis of their lower Ccr2 expression among gated CD16^+^ events [9]. Hence, the proper detection of nonclassical monocytes in BM appears debatable and may indeed result from “contamination” with variable amounts of peripheral blood during the BM aspiration.

In contrast to the Ccr2^++^ classical and intermediate subsets in the periphery, the nonclassical monocytes are Cx3cr1^++^ but dimly express Ccr2 and function mainly as weak phagocytes that patrol the microvasculature in LFA-1 dependent manner to sense pathogens or cell remnants at steady-state [18]. Moreover, nonclassical monocytes express the angiopoietin receptor Tie2 and are further considered as essential paracrine players in tumor angiogenesis [5], [19]. Similar pro-angiogenic characteristics were recently described for the intermediate subset as well [8]. Since the CD14^++^CD16^+^ BMCs express similar levels of Cx3cr1 and display pro-angiogenic properties, it seems that nonclassical monocytes could arise from this mixed population as well but specialize first in the periphery after coming across pathogens at homeostatic conditions, *e.g.* during patrolling the microvasculature of gut and lung.

In summary, a common CD14^++^CD16^+^ monocyte pool featuring mostly intermediate monocytes is primary enriched in human BM but depleted in peripheral blood where this subset exists at lowest percentage. The medullar monocytes seems to require Ccr2, Cxcr4 and possibly CD44 for mobilization and may give rise to more specialized peripheral Ccr2^++^ classical monocytes with implication during inflammation next to Cx3cr1^++^ nonclassical monocytes that are rather recruited at steady-state. The maturation of medullar monocytes obviously associates with decrease in the expression of Cxcr4 and c-kit. Thus, it is conceivable that human monocytes acquire their differential CD14/CD16 signature first in the bloodstream.

## Supporting Information

Figure S1 **Representative histogram overlays for surface markers of the BD Lyoplate screening panel.**
(TIF)

Table S1 **Specific MFI values of chemokine receptor expression on medullar (n = 6) and blood monocyte subsets (n = 10).**
(DOCX)
