# Supplementary figures and images for: Characterization of the CD14++CD16+ Monocyte Population in Human Bone Marrow
Source: PLoS One. 2014 Nov 4;9(11):e112140. doi: 10.1371/journal.pone.0112140 (PMC4219836; doi:10.1371/journal.pone.0112140)

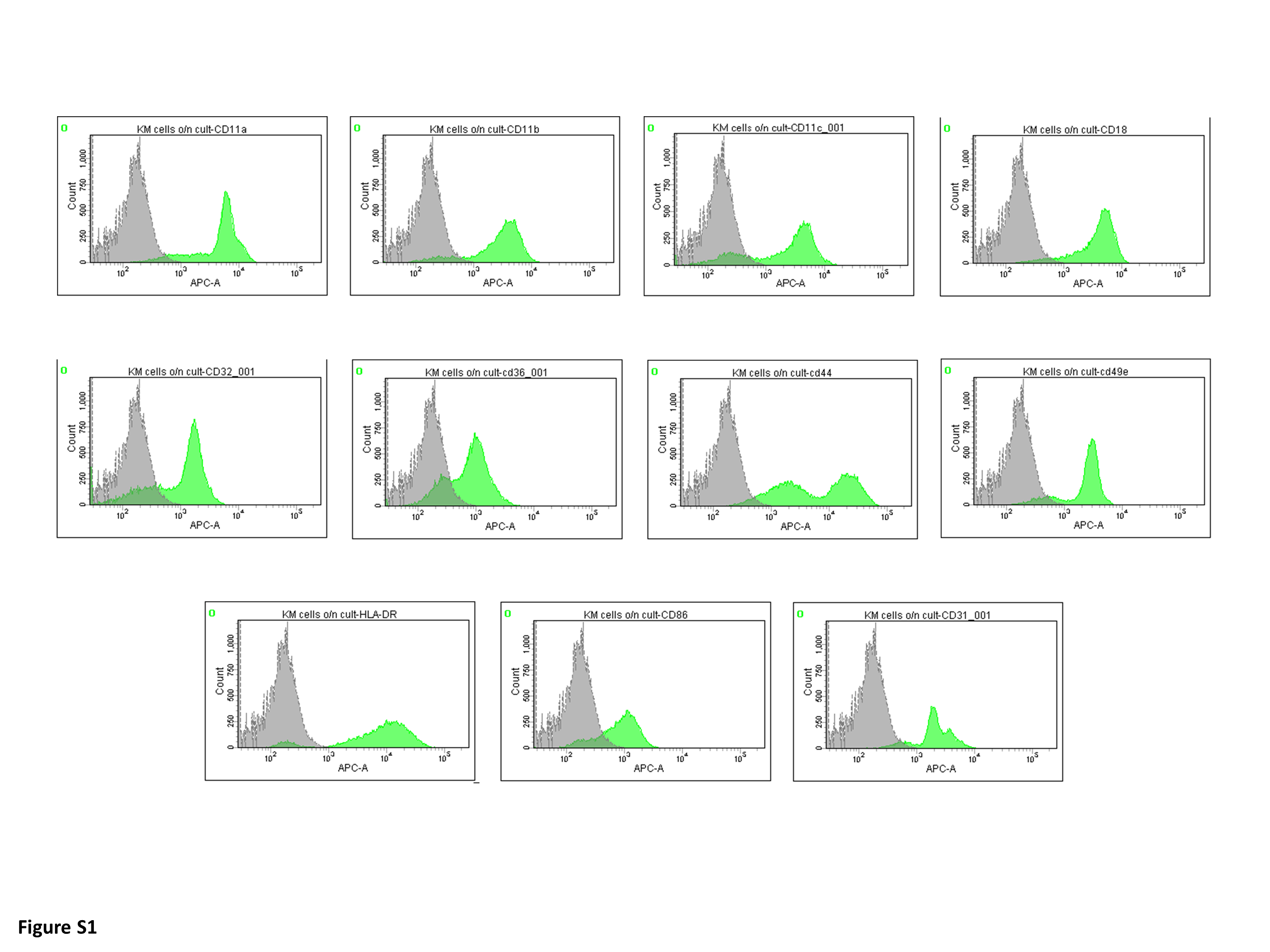

Supplement: Figure S1 — Representative histogram overlays for surface markers of the BD Lyoplate screening panel. (TIF) [file pone.0112140.s001.tif]
