# Supplementary material for: Characterization of the CD14++CD16+ Monocyte Population in Human Bone Marrow
Source: PLoS One. 2014 Nov 4;9(11):e112140. doi: 10.1371/journal.pone.0112140 (PMC4219836; doi:10.1371/journal.pone.0112140)
Supplement: Table S1 — Specific MFI values of chemokine receptor expression on medullar (n = 6) and blood monocyte subsets (n = 10). (DOCX) [file pone.0112140.s002.docx]

**Table S1.** Specific MFI values of chemokine receptor expression on medullar (n=6) and blood monocyte subsets (n=10).

| **Receptor** | **thawed medullar** | **thawed blood** | | | **fresh blood** | | |
| --- | --- | --- | --- | --- | --- | --- | --- |
|  | **CD14^++^CD16^+^** | **CD14^++^CD16^-^** | **CD14^++^CD16^+^** | **CD14^+^CD16^++^** | **CD14^++^CD16^-^** | **CD14^++^CD16^+^** | **CD14^+^CD16^++^** |
| **Ccr2** | 3430±1325 | 3587±1962 | 4203±2405 | 1723±776 | 5948±3827 | 2980±1700 | 310±217 |
| **Ccr5** | 3316±1680 | 2234±459 | 3443±1767 | 1316±614 | 1098±295 | 1413±340 | 658±312 |
| **Cx3cr1** | 4215±2033 | 2665±869 | 4677±1669 | 4818±2086 | 975±472 | 2431±1281 | 3959±2039 |
| **Cxcr2** | 526±230 | 724±145 | 736±247 | 471±107 | 1700±407 | 660±225 | 407±126 |
| **Cxcr4** | 8832±4010 | 5558±2069 | 4970±2408 | 2913±1726 | 2725±951 | 2608±1270 | 3357±881 |
